# Buccal carcinoma associated with adult congenital pulmonary hypoplasia: A case report and literature review

**DOI:** 10.1097/MD.0000000000032142

**Published:** 2022-12-02

**Authors:** Miao Qiu, De-Rong Lin

**Affiliations:** a Department of Tumor Radiotherapy, Shaoxing Second Hospital, Shaoxing, China.

**Keywords:** adult, bronchiectasis, congenital, pulmonary hypoplasia

## Abstract

Congenital pulmonary hypoplasia is a relatively rare lung malformation. The disease is usually identified in the neonatal period or early childhood. Most affected patients die of respiratory insufficiency after birth. Mild congenital dysplasia is asymptomatic, while moderate congenital dysplasia manifests as respiratory distress or does not become evident until adulthood. Cases of congenital pulmonary hypoplasia in adults are rare, especially in combination with malignant tumors.

**Patient concerns:**

We report the case of a 64-year-old man with buccal carcinoma who was accidentally found to have hypoplasia of the left lung during treatment.

**Diagnoses:**

After chest computed tomography and chest radiograph, the diagnosis of adult congenital pulmonary hypoplasia was confirmed.

**Interventions:**

Since the patient had a history of bronchiectasis for more than 30 years and only had 1 healthy lung, the cardiopulmonary compensatory function was poor and the patient could not tolerate surgery, he was given radiotherapy and chemotherapy.

**Outcomes:**

Radiotherapy and chemotherapy were successfully completed, and within the patient’s tolerance, there was no serious adverse reaction of respiratory system.

**Conclusion:**

Congenital pulmonary hypoplasia’s diagnosis is challenging in adults because the condition can easily be mistaken for a more common disease. However, early diagnosis is very important to enable prompt therapy and ensure proper follow-up to detect and treat complications such as pulmonary infection and pulmonary hypertension in a timely manner.

## 1. Introduction

Congenital pulmonary hypoplasia is a relatively rare lung malformation caused by the development of obstructive lung tissue during the embryonic stage. The disease is usually identified in the neonatal period or early childhood. Most affected patients die of respiratory insufficiency after birth. Few cases are diagnosed in adulthood. In this report, we describe the case of a 64-year-old man with buccal cancer who was incidentally diagnosed with adult congenital pulmonary hypoplasia and review the relevant literature.

## 2. Case report

**Figure 1.**
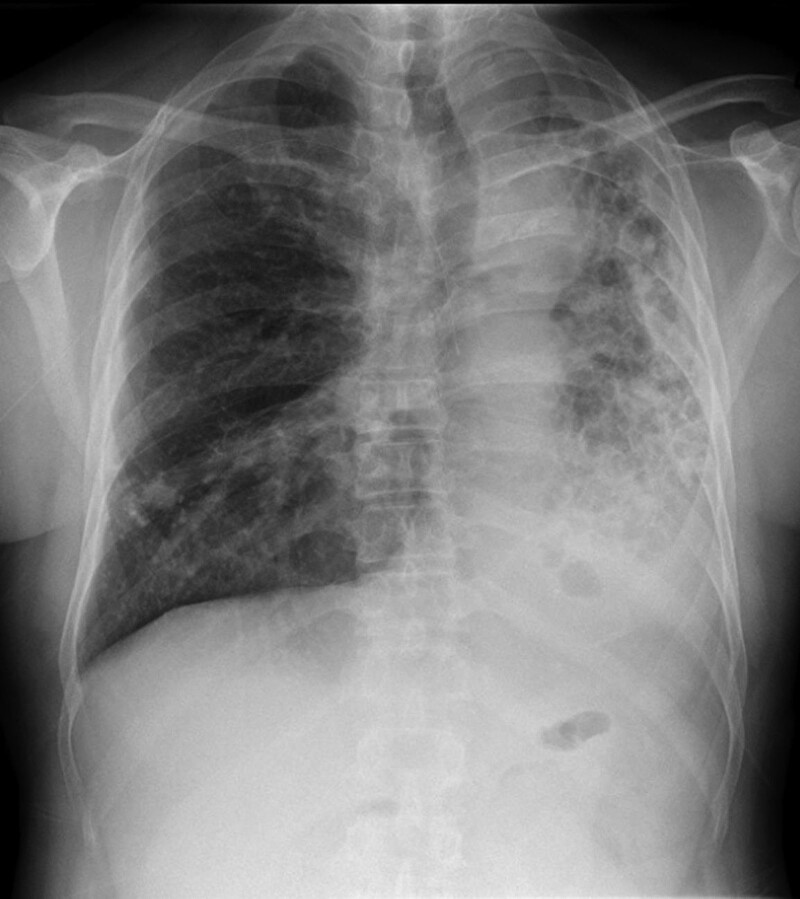
Chest radiograph image showing a decreased volume of the left lung, which was significantly smaller than the right lung.

**Figure 2.**
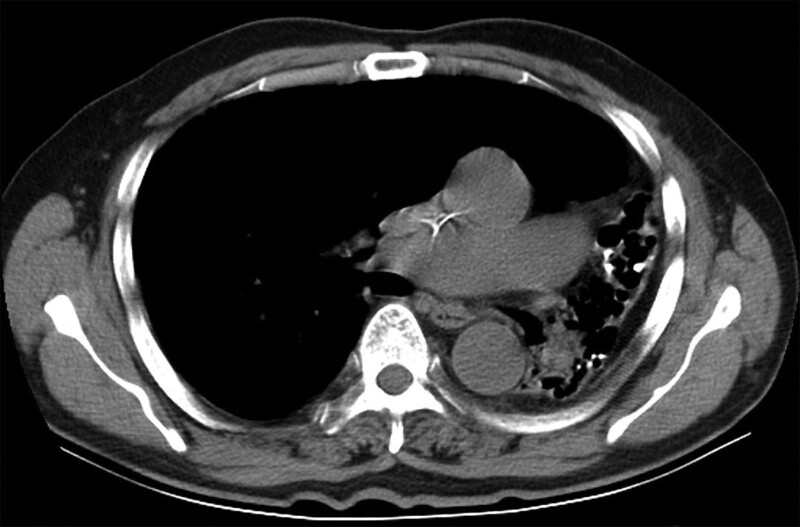
Chest CT image showing significantly decreased volume and bronchiectasis of the left lung corresponding to pulmonary hypoplasia and compensatory expansion of the volume of the right lung. CT = computed tomography.

**Figure 3.**
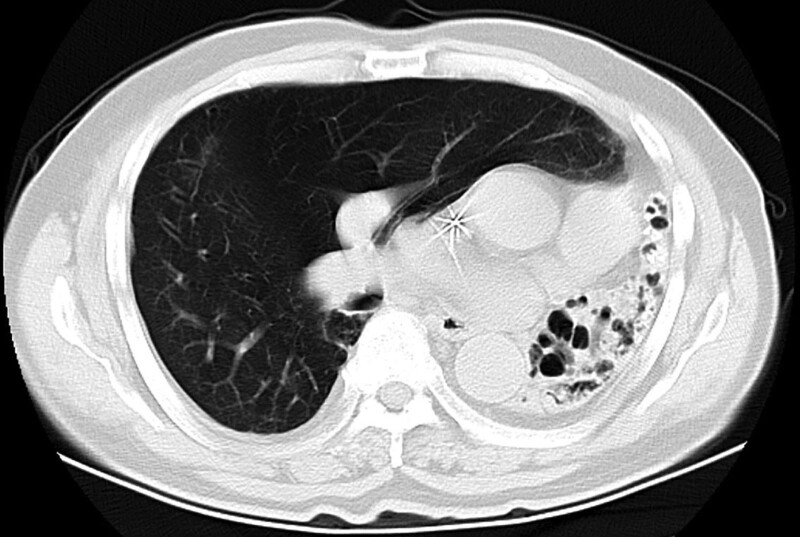
CT scan of the lung window showing the mediastinum and heart shifted to the left with hyperinflation and expansion of the right lung. The left lung is almost entirely hypoplastic with multiple dilated bronchi and a cystic morphology. CT = computed tomography.

A 64-year-old man visited our hospital with a 1-year history of right submaxillary mass and 1-month history of an enlarged right oral mass. Computed tomography (CT) of the oropharynx showed soft tissue nodules under the right mandible, and a biopsy of the right buccal mass was performed. The postoperative pathology showed (right buccal) squamous cell carcinoma. As the patient had a self-reported history of bronchiectasis for more than 30 years, had poor cardiopulmonary compensatory function, and could not tolerate surgery, radiotherapy was recommended. Radiation was delivered at 2 Gy/d for 5 d/wk for 30 fractions; the total dose was 60 Gy. Three cycles of chemotherapy regimen (docetaxel 120 mg day1 + cisplatin 40 mg day1–3) were completed. A chest radiograph showed that the left lung was significantly narrowed (Fig. 1). Chest CT revealed decreased volume of the left lung, multiple bronchiectasis in the lung, compensatory increase in the volume of the right lung, scattered patchy and strip-shaped increased density shadows in the right lung, and dense nodules with a size of about 18 mm × 14 mm in the lower lobe of the right lung. The mediastinum and heart shadow were shifted to the left, and the left pleura was slightly thickened (Figs. 2 and 3). The imaging diagnosis revealed that the left lung was damaged, the right lung had areas of scattered chronic inflammation, and the lower lobe of the right lung was a dense nodule. The family history was positive for congenital left lung hypoplasia in the patient’s mother.

## 3. Discussion

Congenital pulmonary hypoplasia, a rare congenital disease, is a developmental disorder of the pulmonary vessels and bronchus caused by congenital defects of the germ protoplasm or embryoblasts. The prevalence of unilateral pulmonary hypoplasia is 1 to 2 per 12,000 newborns.^[1]^ It is more common in newborns and children and rarely diagnosed in adults. It is characterized by recurrent or incurable pulmonary infections. The etiology of congenital pulmonary hypoplasia is currently unclear but has been hypothesized to be associated with genetic factors or the expectant mother’s contracting rubella infection during the first trimester of pregnancy.^[2]^ Recent studies have shown that it is also related to vitamin A deficiency in the mother.^[3]^ In our case, the patient’s mother also had congenital left lung hypoplasia. This condition can be unilateral or bilateral; in 1955, it was classified by Boyden into 3 categories: pulmonary agenesis (carina, main bronchi, lung tissue, and vascular structures are absent); pulmonary aplasia (a pouch-like, blind-ending main bronchus and carina are present); and pulmonary hypoplasia (destroyed bronchial structures cause maldevelopment of the alveolar tissue, and lung tissue appears as a mediastinal structure).^[4]^ The chest CT of our patient showed a small amount of lung tissue and left bronchus (Fig. 2), indicating that it was type iii.

Patients with congenital pulmonary hypoplasia have no specific clinical manifestations but experience recurrent pulmonary infection, chest tightness, and shortness of breath. Our patient had a 30-year history of bronchiectasis but no obvious symptoms or signs on imaging examinations. The affected thorax is generally normal in the early stage; as the patient grows and develops, the healthy lung tissue expands and fills the affected thorax, resulting in displacement of the mediastinum to the affected side. Due to compensatory pulmonary function on the healthy side, the early clinical symptoms of patients with unilateral pulmonary hypoplasia are often mild, consisting of an occasional cough or mild pulmonary infection. However, the clinical symptoms gradually worsen with age, and about 44% of patients have comorbid pulmonary hypertension.^[5]^ Unilateral pulmonary hypoplasia is most often noted on the left side,^[6]^ as in the current case. As there are 3 lobes in the right lung, compensation is easier, possibly explaining the better survival rate of patients with left lung hypoplasia.^[7]^

The diagnosis of pulmonary hypoplasia can be easily missed if only a chest radiograph is performed. Our patient was diagnosed with bronchiectasis and left atelectasis only because of a chest radiograph that was performed previously when he sought care for a respiratory disease; at that time, left lung hypoplasia was not identified. CT has the advantages of continuous and rapid scanning, volume data acquisition, excellent multi-axial planes, and 3-dimensional image reconstruction. It can display the structures of pulmonary vessels and bronchus, is easy to use and, has high accuracy. Therefore, it is considered the first choice for the diagnosis of congenital pulmonary hypoplasia.^[8]^ The typical CT findings are significantly reduced lung volume on the affected side, increased tissue density, a narrow or blocked bronchial opening, compensated emphysema on the healthy side, and shifting of the mediastinum and heart shadow to the affected side.

This case of bronchiectasis caused by repeated infection during youth was caused by pulmonary hypoplasia. Failure to provide effective treatment resulted in the gradual aggravation of left lung infection and, ultimately, led to lung damage. Because the right lung compensated for the deficiency, further compensatory space was lacking. Considering that the patient was not a candidate for surgical procedures, surgical resection of the buccal cancer was not undertaken. Instead, he was treated with radiotherapy and chemotherapy.

Congenital pulmonary hypoplasia is characterized by no obvious symptoms in the absence of a respiratory infection, necessitating no treatment. Hypoplasia of the alveolar tissue leads to slight fibrosis and a nonfunctional lung accompanied by the lack of relevant surfactants and impaired mucociliary clearance.^[7]^ Secretions easily accumulate in the hypoplastic bronchus, becoming the main pathogenic focus of recurrent respiratory tract infections resulting in delayed infection.

Treatment in adults is usually conservative and includes regular monitoring, treatment for the infection, use of bronchodilators or expectorants to alleviate symptoms, and preventive vaccination. In some patients, abnormal airway angle, defective mucociliary clearance, and the accumulation of blind dysplastic secretions lead to repeated airway infection and cystic degeneration, which are indications for surgical resection of the affected lung.^[9,10]^

## 4. Conclusion

Although congenital pulmonary hypoplasia is rare and usually detected in childhood, it can manifest in adults in special circumstances since the combination of certain characteristics may enable patients to achieve long-term survival. Its diagnosis is challenging in adults because the condition can easily be mistaken for a more common disease. However, early diagnosis is very important to enable prompt therapy and ensure proper follow-up to detect and treat complications such as pulmonary infection and pulmonary hypertension in a timely manner. Patients with obvious symptoms and no severe pulmonary impairment require surgical resection of the affected lung.

## Author contributions

**Investigation:** De-Rong Lin.

**Writing – original draft:** Miao Qiu.

**Writing – review & editing:** Miao Qiu.

## References

[1] Kant S. Unilateral pulmonary hypoplasia – a case report. Lung India. 2007;24:69–71.

[2] Tang JS Kauffman SL Lynfield J. Hypoplasia of the pulmonary arteries in infants with congenital rubella. Am J Cardiol. 1971;27:491–6. 5552090 10.1016/0002-9149(71)90411-5

[3] Baptista MJ Melo-Rocha G Pedrosa C. Antenatal vitamin A administration attenuates lung hypoplasia by interfering with early instead of late determinants of lung underdevelopment in congenital diaphragmatic hernia. J Pediatr Surg. 2005;40:658–65. 15852274 10.1016/j.jpedsurg.2005.01.034

[4] Boyden EA. Developmental anomalies of the lungs. Am J Surg. 1955;89:79–89. 13218221 10.1016/0002-9610(55)90510-9

[5] Harkel A Blom NA Ottenkamp J. Isolated unilateral absence of a pulmonary artery: a case report and review of the literature.. Chest. 2002;122:1471–7. 12377882 10.1378/chest.122.4.1471

[6] Reading David W Oza U. Unilateral absence of a pulmonary artery: a rare disorder with variable presentation. Proceedings (Baylor University, Medical Center). 2012;25:115–8. 22481838 10.1080/08998280.2012.11928802 PMC3310505

[7] Katsenos S Antonogiannaki EM Tsintiris K. Unilateral primary lung hypoplasia diagnosed in adulthood. Resp Care. 2014;59:e47–50. 10.4187/respcare.02740 24026186

[8] Beigelman C Howarth NR Chartrand-Lefebvre C. Congenital anomalies of the tracheobronchial branching patterns: spiral CT aspects in adults. Eur Radiol. 1998;8:79–85. 9442135 10.1007/s003300050343

[9] Thomas RJ Lathif HC Sen S. Varied presentations of unilateral lung hypoplasia and agenesis: a report of four cases. Pediatr Surg Int. 1998;14:94–5. 9880709 10.1007/s003830050447

[10] Laberge JM Puligandla P Flageole H. Asymptomatic congenital lung malformations. Semin Pediatr Surg. 2005;14:16–33. 15770585 10.1053/j.sempedsurg.2004.10.022

